# Dual-state six-channel polarization multiplexing in reconfigurable metasurfaces

**DOI:** 10.1515/nanoph-2025-0403

**Published:** 2025-11-10

**Authors:** Sujun Xie, Tianxu Jia, Xiaoyue Ma, Bingjue Li, Ruohu Zhang, Zhigang Li, Binfeng Yun, Hyeonsu Heo, Nara Jeon, Guanghao Rui, Junsuk Rho

**Affiliations:** Department of Optical Engineering, School of Electronic Science and Engineering, Southeast University, Nanjing 211189, Jiangsu, China; Department of Mechanical Engineering, Pohang University of Science and Technology (POSTECH), Pohang 37673, Republic of Korea; School of Electrical and Information Engineering, Tianjin University, Tianjin 300072, China; School of Mechanical Engineering, Southeast University, Nanjing 211189, Jiangsu, China; Department of Chemical Engineering, POSTECH, Pohang 37673, Republic of Korea; Department of Electrical Engineering, POSTECH, Pohang 37673, Republic of Korea; POSCO-POSTECH-RIST Convergence Research Center for Flat Optics and Metaphotonics, Pohang 37673, Republic of Korea

**Keywords:** polarization multiplexing, phase change materials, dynamic metasurface

## Abstract

Dynamically tunable metasurfaces based on phase-change materials (PCMs) have become important platforms for realizing reconfigurable optical systems. Nevertheless, achieving multiple independent functionalities within a single device, particularly under polarization multiplexing, remains difficult due to limited design flexibility. In this study, we present a metasurface design framework that reaches the theoretical maximum of six independent phase modulation functions by simultaneously controlling the polarization states and the crystallinity of the PCM. This is implemented through a pixel-extension strategy, where each nanofin functions independently in amorphous state and is reorganized into superpixels with distinct optical responses in crystalline state. To support this, a forward filtering algorithm is developed to efficiently determine structural configurations under dual-state constraints. The effectiveness of the proposed approach is confirmed through two representative implementations, including dynamically switchable multifocal metalenses and multichannel holography. In addition, a progressive encoding strategy is introduced, which deliberately utilizes inter-state crosstalk to hierarchically embed optical information across material states. This compact and reconfigurable metasurface platform offers high functional density and flexible control, holding strong potential for applications in optical communication, information encryption, and adaptive display technologies.

## 1 Introduction

Metasurfaces [1], [2], [3], composed of planar arrays of subwavelength nanostructures, have been widely regarded as a viable alternative to traditional bulk optical components due to their ultrathin profile, compatibility with established microfabrication processes, and potential for compact integration. Through the precise geometric design of constituent elements, metasurfaces are capable of controlling the phase [4], [5], amplitude [6], [7], and polarization [8], [9] of light, thereby enabling diverse applications in high-resolution imaging [10], [11], [12], miniaturized optical communication systems [13], [14], sensitive optical sensing [15], [16], optical neural computing [17], [18], and structured light generation [19], [20], [21].

To further advance metasurface technology and broaden its practical applicability, it is essential to incorporate multiple optical functionalities within a single device. This objective has stimulated the development of multiplexing metasurfaces, including schemes based on polarization [22], [23], [24], [25], wavelength [26], [27], incident angle [28], [29], and orbital angular momentum [30], [31], all of which allow for simultaneous control over multiple independent optical channels. Among these strategies, polarization multiplexing has attracted particular interest due to its relevance for high-capacity information processing. Independent phase modulation of two orthogonal polarization channels has been demonstrated through the combined use of geometric and propagation phase [32]. Additionally, a structural mapping method has been proposed to achieve independent control of three orthogonal polarization states via three geometric parameters [33]. However, planar metasurfaces are fundamentally constrained to supporting no more than three independent polarization channels [34]. Although attempts have been made to extend this capacity through engineered noise [35] or advanced optimization algorithms [36], [37], such approaches inevitably result in inter-channel crosstalk. Furthermore, the majority of polarization multiplexing metasurfaces remains static once fabricated, with dynamic functionality being restricted to variations in the input polarization state alone. These limitations hinder the full exploitation of metasurfaces’ potential for reconfigurable photonic systems.

To address these constraints, dynamically tunable metasurfaces based on active platforms have been extensively explored [38], [39]. Among them, phase-change materials (PCMs) have attracted considerable attention, as they exhibit reversible and nonvolatile transitions between amorphous and crystalline states, accompanied by significant changes in refractive index [40]. These transitions can be induced by external stimuli such as optical, electrical, or thermal input, and their cycling stability can exceed one trillion switching events [41], [42]. By employing the material crystallinity as an additional degree of freedom, the design space for polarization multiplexing can be significantly expanded. Several recent advances have illustrated this potential. A three-channel metalens was realized by jointly modulating crystallinity and polarization using a tellurium-based alloy (GSST) [43]. Direction-dependent holography was demonstrated via Janus metasurfaces incorporating vanadium dioxide (VO_2_) [44]. Four-channel holography was achieved by applying a modified iterative design algorithm to metasurfaces composed of antimony trisulfide (Sb_2_S_3_) [45], and a bilayer structure based on VO_2_ was reported to support dual tripolarization holographic states, thereby enabling six distinct functionalities [46]. Despite these advances, current PCM-based single-layer metasurfaces still fall short of achieving the theoretical maximum of six independent functionalities, which would result from the combination of three polarization channels and two material states. The limited degrees of freedom and inherent structural coupling prevent conventional designs from reaching this theoretical maximum.

In this work, we propose a metasurface design strategy that enables six independent phase modulation functions within a single-layer device. This is realized by combining dual-state control of phase-change materials with polarization multiplexing, which significantly expands the available design space. The proposed method employs a pixel-extension strategy that differentiates effective pixels between the amorphous and crystalline states, together with a forward filtering algorithm that guides efficient structural selection under multiple constraints. The effectiveness of this strategy is demonstrated through the implementation of a dynamically reconfigurable multifocal metalens and a six-channel holographic metasurface. Furthermore, the commonly undesired inter-state crosstalk is intentionally utilized to enable progressive information encoding across material states. This work represents, to the best of our knowledge, the first realization of six fully independent functionalities in a single-layer metasurface through combined control of polarization and crystallinity of PCM, which presents potential for applications in optical communication, information encryption, and adaptive display technologies.

## 2 Principles and methods

**Figure 1:**
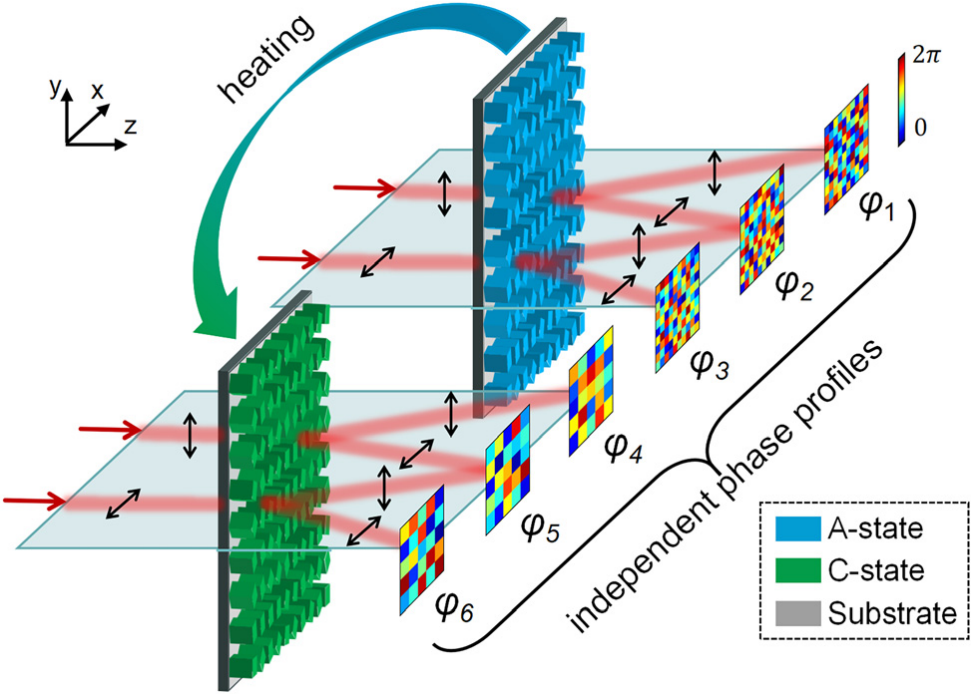
Schematic illustration of the proposed reconfigurable multifunctional metasurface, which enables independent phase modulation of both co-polarized and cross-polarized components of incident linearly polarized light under both the A- and C-state of the phase-change material.

The conceptual framework of the proposed reconfigurable multifunctional metasurface is illustrated in Figure 1. By tailoring both the polarization states of the incident and transmitted waves, as well as the crystallinity state of the PCM, six independent phase modulations can be achieved. Specifically, in the amorphous state (A-state), the transmitted *y*- (or *x*-) polarized component of an *x*- (or *y*-) polarized incident wave experiences a phase modulation of exp(*φ*_2_). Additionally, *x*- and *y*-polarized incident waves yield co-polarized transmitted components modulated by exp(*iφ*_1_) and exp(*iφ*_3_), respectively. The corresponding phase profiles are denoted by *φ*_1_, *φ*_2_ and *φ*_3_. Upon transitioning to the crystalline state (C-state), a distinct set of phase profiles (*φ*_4_, *φ*_5_, and *φ*_6_) is supported in the same polarization channels. Notably, the number of effective pixels is reduced in the C-state, due to a change in the nanofin grouping strategy, which will be discussed in detail later.

For a single-layer metasurface, the optical response at each pixel can be represented by a 2 × 2 Jones matrix

(1) $$\begin{array}{c} J=\left[\begin{array}{cc} T_{xx} & T_{xy}\\ T_{yx} & T_{yy} \end{array}\right], \end{array}$$

where each element corresponds to a unique mapping from input to output polarization with a complex coefficient $T_{ij}=t_{ij}{\text{e}}^{\text{i}\varphi_{ij}}\left(i,j=x,y\right)$ , where *t*_*ij*_ and *φ*_*ij*_ are the amplitude and phase responses from *j*-polarized input to *i*-polarized output. Due to the inherent in-plane symmetry of the metasurface structure, the off-diagonal terms must be equal (i.e., *T*_*yx*_ = *T*_*xy*_). The relationship between the incident and transmitted electric fields is given by *E*_out_ = *J* · *E*_in_ where *E*_in_ = [1, 0]^T^ or [0, 1]^T^ corresponds to *x*- or *y*-polarized incident light, respectively. The resulting transmitted fields become *E*_out_ = ${\left[t_{xx}{\text{e}}^{\text{i}\varphi_{xx}},\;t_{xy}{\text{e}}^{\text{i}\varphi_{xy}}\right]}^{\text{T}}$ or ${\left[t_{xy}{\text{e}}^{\text{i}\varphi_{xy}},\;t_{yy}{\text{e}}^{\text{i}\varphi_{yy}}\right]}^{\text{T}}$ , respectively. This indicates that the co-polarized transmitted components acquire phase shifts of *φ*_*xx*_ and *φ*_*yy*_, while the cross-polarized component undergoes a phase modulation of *φ*_*xy*_. Therefore, to achieve independent phase control in the three polarization channels (*x* → *x*, *x* → *y*/*y* → *x*, *y* → *y*), the phase of each complex element in the Jones matrix must be independently tunable.

Rectangular PCM nanofins are employed as the fundamental unit cells of the metasurface, and their ability to control polarization arises from local birefringence and geometric rotation. In the A-state, the action of a nanofin on incident light is described by *J* = *R*(−*θ*) ⋅ Λ ⋅ *R*(*θ*), where *R*(*θ*) and Λ are the rotation matrix and the birefringent Jones matrix, respectively:

(2) $$\begin{array}{c} R\left(\theta \right)=\left[\begin{array}{cc} \cos\theta & \sin\theta \\ -\sin\theta & \cos\theta \end{array}\right],\;\Lambda =\left[\begin{array}{cc} T_{x}^{\text{A}} & 0\\ 0 & T_{y}^{\text{A}} \end{array}\right], \end{array}$$

where *θ* denotes the nanofin rotation angle with respect to the *x*-axis, $T_{i}^{\text{A}}=t_{i}^{\text{A}}\varphi_{i}^{\text{A}}\left(i=x,y\right)$ are the transmission coefficients along the fast and slow axes, assumed to align with the *x*- and *y*-axes when *θ* = 0°. Substituting (Eq. (2)) into the nanofin’s Jones matrix yields:

(3) $$\begin{array}{c} J_{\text{A}}=\left[\begin{array}{cc} T_{x}^{\text{A}}\cos^{2}\theta +T_{y}^{\text{A}}\sin^{2}\theta & \left(T_{x}^{\text{A}}-T_{y}^{\text{A}}\right)\sin\theta \cos\theta \\ \left(T_{x}^{\text{A}}-T_{y}^{\text{A}}\right)\sin\theta \cos\theta & T_{x}^{\text{A}}\sin^{2}\theta +T_{y}^{\text{A}}\cos^{2}\theta \end{array}\right]. \end{array}$$

Using this formulation, we show that for any target set of phases (*φ*_1_, *φ*_2_, *φ*_3_) ∈ [–*π*, *π*], there exists a corresponding set of parameters ( $T_{x}^{\text{A}},T_{y}^{\text{A}},\theta$ ) satisfying:

(4) $$\begin{array}{c} \left\{\begin{array}{c} \varphi_{1}=\arg\left(T_{x}^{\text{A}}\cos^{2}\theta +T_{y}^{\text{A}}\sin^{2}\theta \right),\\ \varphi_{2}=\arg\left[\left(T_{x}^{\text{A}}-T_{y}^{\text{A}}\right)\sin\theta \cos\theta \right],\;\\ \varphi_{3}=\arg\left(T_{x}^{\text{A}}\sin^{2}\theta +T_{y}^{\text{A}}\cos^{2}\theta \right). \end{array}\right. \end{array}$$

As demonstrated in Section 1 of the Supporting Information, a solution to Eq. (4) always exists, indicating that independent phase modulation in all three polarization channels can be achieved by adjusting the three parameters $\left(T_{x}^{\text{A}},T_{y}^{\text{A}},\theta \right)$ .

When the PCM nanofins are transformed into the C-state by external stimuli, the orientation *θ* remains unchanged, while the birefringent properties evolve. The transmission coefficients transition from $\left(T_{x}^{\text{A}},T_{y}^{\text{A}}\right)$ to $\left(T_{x}^{\text{C}},T_{y}^{\text{C}}\right)$ , the superscript indicates different crystallinity states of the material. These two additional parameters increase the degrees of freedom. However, if each nanofin is continuously regarded as an individual pixel, the number of available degrees of freedom in the C-state remains insufficient to satisfy the constraints associated with independent phase modulation across three polarization channels. Specifically, since only two structural parameters are adjustable, whereas three independent phase equations must be fulfilled. Actually, it becomes feasible to modulate only one single polarization channel in this state (refer to Section 2 of the Supporting Information).

**Figure 2:**
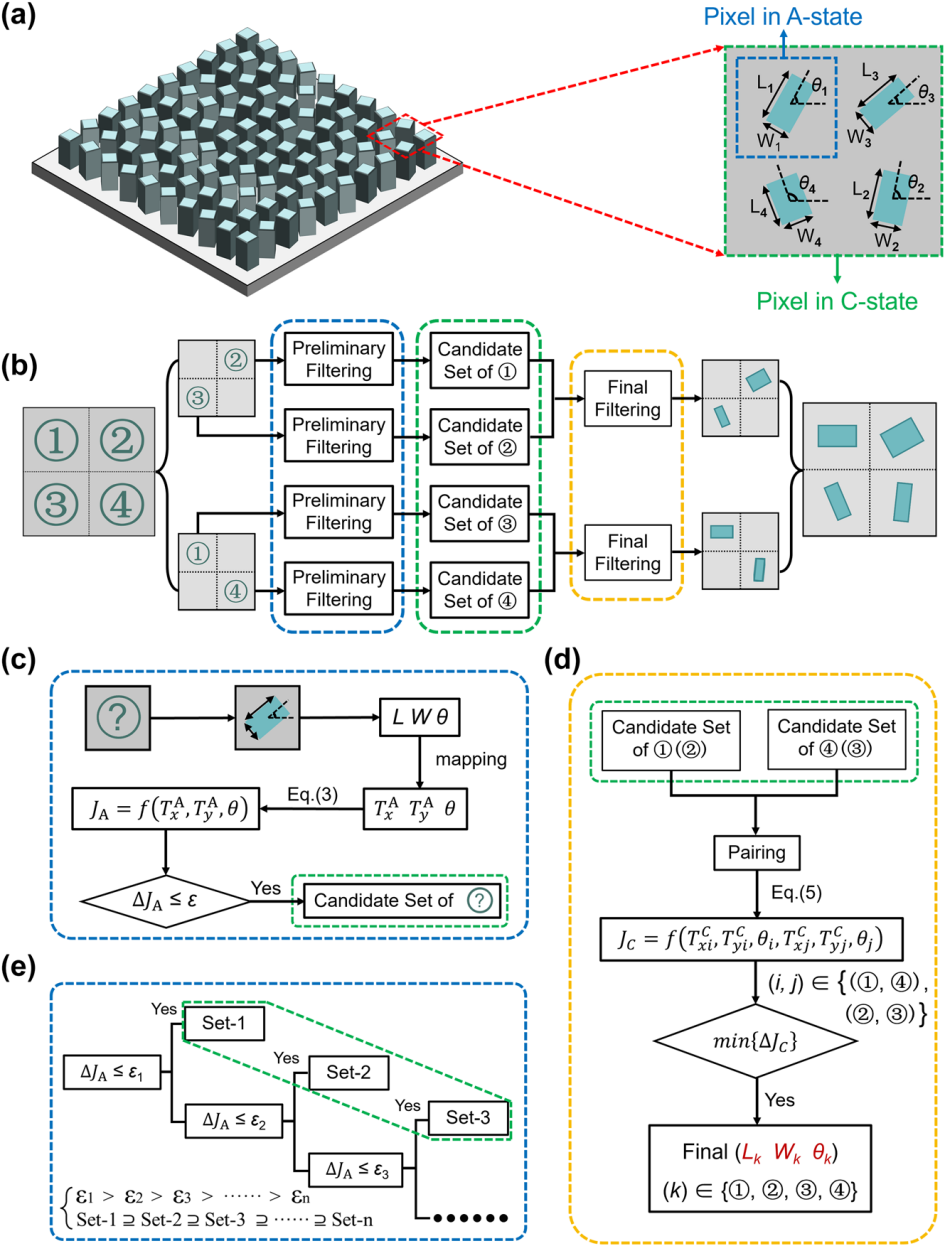
Design concept and corresponding implementation strategy of the proposed metasurface. (a) Structural configuration of individual metasurface pixels in the A- and C-state, illustrating the transition from single-pixel to superpixel representation. The “pixel in C-state” corresponds to the overlap of two superpixels (i.e., two sets of diagonally adjacent nanofins) that share the same pixel region. (b) Schematic overview of the forward design framework, which systematically determines suitable nanofin geometries under dual-state constraints. (c) Preliminary filtering process that selects candidate configurations enabling accurate phase modulation in the A-state. (d) Final filtering process that evaluates all possible configuration pairs to ensure the desired phase modulation is simultaneously satisfied in both A- and C-states. (e) Multi-threshold filtering scheme designed to balance phase errors between the two material states. In (c)–(e), blue dashed boxes denote the preliminary filtering stage, green regions represent the candidate configuration sets, and yellow highlights indicate the final selection outcome.

To address this limitation, a pixel extension design strategy is proposed during the metasurface design in the C-state. As illustrated in Figure 2a, each nanofin functions independently in the A-state, with its Jones matrix *J*_A_ calculated via (Eq. (3)). In contrast, two diagonally adjacent nanofins are grouped into a superpixel in the C-state, whose overall Jones matrix *J*_C_ is given by the coherent sum of the individual Jones matrices of the two nanofins:

(5) $$\begin{array}{c} J_{\text{C}}=\overset{2}{\underset{k=1}{\sum }}J_{\text{C}k}=\overset{2}{\underset{k=1}{\sum }}R\left(-\theta_{k}\right)\left[\begin{array}{cc} T_{xk}^{\text{C}} & 0\\ 0 & T_{yk}^{\text{C}} \end{array}\right]R\left(\theta_{k}\right), \end{array}$$

where *J*_C*k*_ (*k* = 1, 2) is the Jones matrix of the *k*th nanofin within the superpixel. Each crystalline nanofin contributes two degrees of freedom, yielding four parameters: $\left(T_{x1}^{\text{C}},T_{y1}^{\text{C}},T_{x2}^{\text{C}},T_{y2}^{\text{C}}\right)$ for tuning *J*_C_. Similarly, according to target set of phases for C-state (*φ*_4_, *φ*_5_, *φ*_6_) ∈ [–*π*, *π*], we can obtain the following equations:

(6) $$\begin{array}{c} \left\{\begin{array}{c} \varphi_{4}=\arg\left(\overset{2}{\underset{k=1}{\sum }}\left(T_{xk}^{\text{C}}\cos^{2}\theta_{k}+T_{yk}^{\text{C}}\sin^{2}\theta_{k}\right)\right),\\ \varphi_{5}=\arg\left(\overset{2}{\underset{k=1}{\sum }}\left(\left(T_{xk}^{\text{C}}-T_{yk}^{\text{C}}\right)\sin\theta_{k}\cos\theta_{k}\right)\right),\\ \varphi_{6}=\arg\left(\overset{2}{\underset{k=1}{\sum }}\left(T_{xk}^{\text{C}}\sin^{2}\theta_{k}+T_{yk}^{\text{C}}\cos^{2}\theta_{k}\right)\right). \end{array}\right. \end{array}$$

The solvability of this equation and the resulting independent phase control are demonstrated in Section 3 of the Supporting Information. This approach effectively compensates for the limited controllability for the metasurface design in the C-state, and the C-state superpixel is designed using two nanofins to achieve independent phase control of three new polarization channels while maximizing pixel density and minimizing optimization complexity. Furthermore, due to weak inter-nanofin coupling, the Jones matrix of the superpixel can be accurately approximated as the sum of its constituent parts [34].

Then, we need to translate the proposed framework into a practical design algorithm. It must be recognized that the birefringence of PCM nanofins in both material states is determined by their geometric dimensions and cannot be tuned independently, once a specific geometry is selected, all relevant optical parameters are simultaneously constrained. Therefore, a forward-design strategy is introduced in Figure 2b. The design process can be divided into two steps. Firstly, a preliminary filtering step is performed, as shown in Figure 2c. For each nanofin with length (*L*) and width (*W*), a set of structural configurations (*L*, *W*, *θ*) is generated. Each configuration is mapped to its corresponding optical parameters $\left(T_{x}^{\text{A}},T_{y}^{\text{A}},\theta \right)$ , which determine the Jones matrix *J*_A_ through (Eq. (3)). The average phase error between *J*_A_ and the target Jones matrix of A-state at the pixel is then evaluated and denoted as Δ*J*_A_. If Δ*J*_A_ is below a predefined threshold *ε*, the configuration (*L*, *W*, *θ*) is retained. All such configurations form the candidate set of that pixel. Secondly, a final filtering step is applied, as shown in Figure 2d. For each pair of diagonally adjacent pixels, one configuration is selected from each of their candidate sets. Their respective C-state parameters $\left(T_{x1}^{\text{C}},T_{y1}^{\text{C}},\theta_{1}\right)$ and $\left(T_{x2}^{\text{C}},T_{y2}^{\text{C}},\theta_{2}\right)$ are used to determine the superpixel Jones matrix *J*_C_ via (Eq. (5)). The phase error between *J*_C_ and the target Jones matrix of C-state is calculated and denoted as Δ*J*_C_. The pair with the minimal Δ*J*_C_ is selected from all possible combinations. In this way, the structural parameters for all nanofins on the metasurface are fully determined. Furthermore, to ensure low design error in both material states, a multi-threshold filtering strategy is introduced, as illustrated in Figure 2e. Instead of applying a uniform threshold across all pixels, multiple candidate sets corresponding to various thresholds are assigned to each pixel. The optimal configuration is selected through global evaluation to ensure satisfactory performance in both the A- and C-states. The proposed two-step forward design algorithm offers a computationally efficient and systematically robust approach for realizing reconfigurable multifunctional metasurfaces. In addition, the global design process of the metasurface is presented in Section 4 of the Supporting Information.

Therefore, the distinct functionalities observed when switching from the A-state to the C-state arise from refractive index modulation of the phase-change material, which modifies nanofin birefringence and reconstructs the Jones matrix elements. Within the forward filtering framework, phase errors in both states are jointly constrained so that the metasurface maps the responses in the A- and C-state to two separate sets of target phases. Switching the material state thus selects between two predesigned wavefronts and enables fully independent optical functions.

## 3 Results and discussion

### 3.1 Design of unit cells

As outlined in the preceding section, the proposed design methodology for the metasurface requires precise control over the birefringent optical responses of nanofins in both the amorphous and crystalline material states. To support this requirement, an optical response database was constructed, in which each entry corresponds to a specific nanofin geometry defined by its length and width, and maps this geometry to the associated phase delay and transmission efficiency under polarized illumination. In order to enable independent and flexible phase modulation, it is required that the achievable phase delays in both material states span the full range from 0 to 2*π*.

To validate the applicability of the proposed design methodology within the visible spectral range, the operating wavelength was fixed at 633 nm, and Sb_2_S_3_ was selected as the phase-change material. This material was chosen due to its favorable properties, including high thermal stability, low optical absorption across the visible spectrum, and a large refractive index contrast between its amorphous and crystalline states. These characteristics collectively render it suitable for the realization of dynamically reconfigurable metasurfaces operating in the visible domain. The refractive index data employed in this study were obtained from previously reported experimental measurements [41].

**Figure 3:**
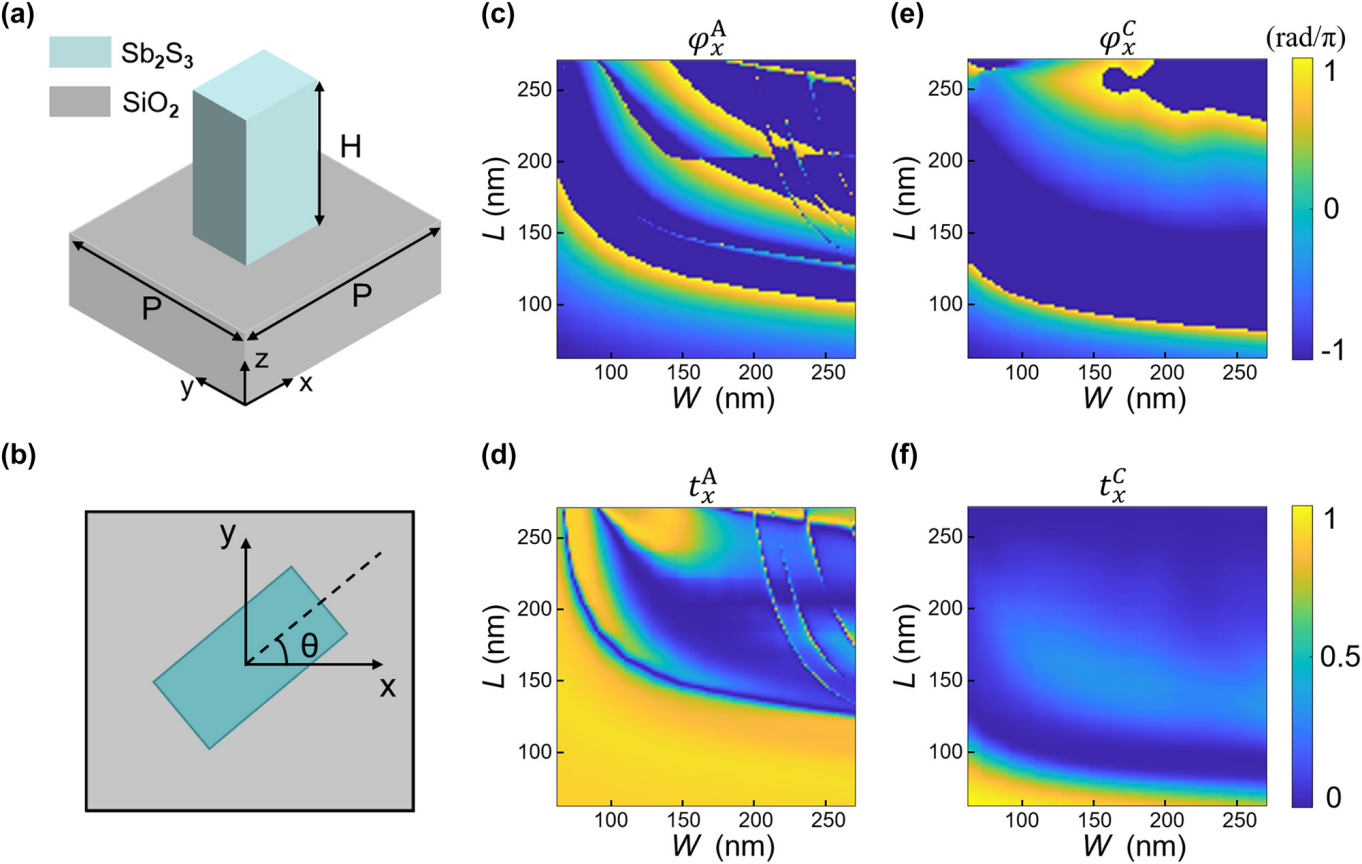
Simulated amplitude and phase responses of the designed Sb_2_S_3_ nanofin. (a) Three-dimensional and (b) top-down views of a single Sb_2_S_3_ nanofin positioned on a glass substrate. (c–f) Numerically calculated amplitude and phase of the transmission coefficients in both the A- and C-state. The length and width of the nanofin are independently swept from 60 nm to 280 nm under *x*-polarized illumination at a fixed wavelength of 633 nm.

The structural design of the unit cell is illustrated in Figure 3a and b, which present the three-dimensional and top-down views, respectively. Each Sb_2_S_3_ nanofin was positioned on a square lattice with a period of 400 nm and a height of 620 nm. Numerical simulations were performed using the finite-difference time-domain method, wherein the phase delay and transmittance were evaluated for different *L*–*W* combinations under *x*-polarized incident light. The resulting data are shown in Figure 3c–f. Owing to the geometric symmetry of the nanofins, the optical response to *y*-polarized illumination can be directly derived by interchanging the coordinate axes of the results.

### 3.2 Dynamic multifocal metalens

**Figure 4:**
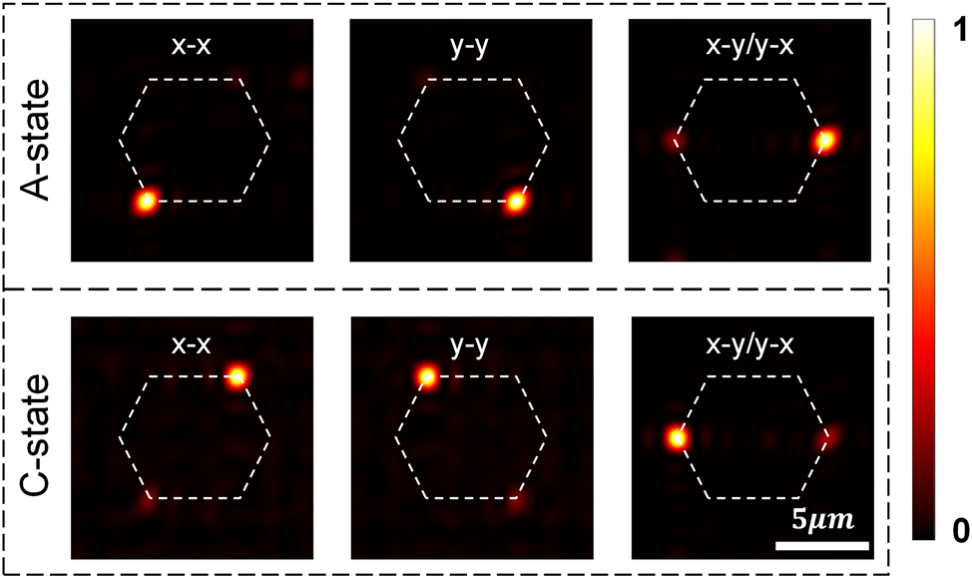
Simulated optical performance of the proposed dynamically reconfigurable multifocal metalens. (a–c) Intensity distributions at the designed focal plane corresponding to three polarization channels (*x*–*x*, *x*–*y*, *y*–*y*) in the A-state. (d–f) Corresponding intensity distributions under the same polarization configurations in the C-state. Six focal spots are preassigned at coordinates (−3 μm, 4 μm), (3 μm, 4 μm), (5 μm, 0 μm), (3 μm, −4 μm), (−3 μm, −4 μm), and (−5 μm, 0 μm), forming a hexagonal pattern indicated by white dashed lines.

To evaluate the functional versatility of the proposed metasurface platform, a dynamically reconfigurable multifocal metalens supporting six independent optical channels was numerically demonstrated. In conventional multifocal metalens configurations, multiple lens segments with distinct focal positions are co-designed within a single plane, which often results in elevated background noise and degraded image contrast. In addition, such systems typically rely on mechanical movement or switching elements to alternate between focal positions, thereby limiting their operational flexibility. In the present design, switching among multiple focal spots is achieved through the coordinated modulation of both the crystallinity of Sb_2_S_3_ and the polarization configuration of the incident and transmitted light. This approach enables dynamic reconfiguration without the need for any physical or mechanical adjustment. A laterally distributed multifocal metalens was selected as a representative example. The phase profile corresponding to the *i*-th focal point, located at $\left(x_{i},y_{i}\right)$ is described by the following expression:

(7) $$\begin{array}{c} \varphi_{i}\left(x,y\right)=-\frac{2\pi }{\lambda }\left(\sqrt{{\left(x-x_{i}\right)}^{2}+{\left(y-y_{i}\right)}^{2}+f^{2}}-f\right), \end{array}$$

where *λ* represents the operating wavelength and the focal length is set to *f* = 80 μm. The metasurface was designed with a total area of 40 μm × 40 μm, consisting of a 100 × 100 array of nanofins, which corresponds to the same number of individually addressable pixels in the A-state. In the C-state, adjacent nanofins are grouped into superpixels, resulting in a reduced array of 2 × 50 × 50 effective pixels. As shown in Figure 4, the focal positions are precisely defined by selecting appropriate combinations of polarization and crystallinity state. The phase profiles corresponding to six distinct focal points are provided in Section 5 of the Supporting Information, demonstrating the capability to realize diverse phase modulation functionalities.

To quantitatively assess the level of optical isolation among the six operational channels, a noise-to-signal ratio, defined as *η* = *I*_noise_/*I*_signal_ was employed, where *I*_signal_ and *I*_noise_ represent the focal intensities at the designated target location and at undesired crosstalk positions, respectively. Two distinct sources of crosstalk were considered in the analysis: the first arising from inter-channel interference between orthogonal polarization states under a fixed crystallinity condition, and the second resulting from residual overlap between the A- and C-state while maintaining a constant polarization configuration. For a fixed material state, the level of inter-polarization crosstalk was consistently observed to remain below 1 % across all channels, a result attributed to the mutual orthogonality of the polarization states involved. This minimal coupling was therefore regarded as negligible background noise. In contrast, more pronounced crosstalk was identified between the A- and C-state configurations, even when the polarization state was held constant. Specifically, when operating in the A-state, residual functionalities associated with the C-state introduced leakage into the *x* → *x*, *x* → *y*, and *y* → *y* channels at intensity levels of 6.9 %, 7.7 %, and 20.8 %, respectively. Conversely, when operating in the C-state, the remaining influence of the A-state gave rise to crosstalk levels of 18.3 %, 20.3 %, and 26.5 % in the corresponding channels. This is primarily attributed to the broadband resonant nature of the employed metasurface elements, which tend to partially retain their phase-modulation characteristics even after undergoing refractive index transitions induced by the material’s crystallinity change. In principle, such effects could be substantially mitigated by adopting nanostructures engineered to exhibit spectrally narrowband and highly state-selective resonance responses. The absolute transmittances of the three channels in the A-state are 41.4 %, 16.8 %, and 41.6 %, with absolute focusing efficiencies of 10.1 %, 5.2 %, and 10.5 %. In the C-state, the values are 14.2 %, 1.6 %, and 14.1 %, with absolute focusing efficiencies of 1.1 %, 0.6 %, and 1.0 %. The efficiency degradation in the C-state primarily originates from the enhanced optical absorption of Sb_2_S_3_ and the reduced number of effective pixels. The metasurface efficiency can be further improved by selecting low-loss operating spectral bands or materials with lower loss at the operating wavelength, increasing the phase-coverage density of the unit-cell library to reduce phase errors, and upgrading the objective function from a single-phase objective to a multi-objective one that incorporates amplitude weighting.

### 3.3 Dynamic holographic imaging

Multichannel holography based on metasurface platforms has been recognized as a promising approach to significantly enhance optical information density, and has attracted considerable attention in applications such as optical encryption and reconfigurable imaging displays. However, within the visible spectral regime, the dynamic reconstruction of multiple holographic channels remains a substantial challenge, primarily due to inherent material limitations and structural design constraints associated with conventional metasurface architectures. To further validate the generality and adaptability of the proposed design framework, a reconfigurable metasurface was numerically demonstrated to enable dynamic switching among six distinct holographic images. The metasurface was composed of an array of 200 × 200 nanopillars, each functioning as an individually addressable unit. The phase distributions corresponding to the six target holograms were determined through a phase retrieval process implemented using the Gerchberg–Saxton (GS) algorithm.

**Figure 5:**
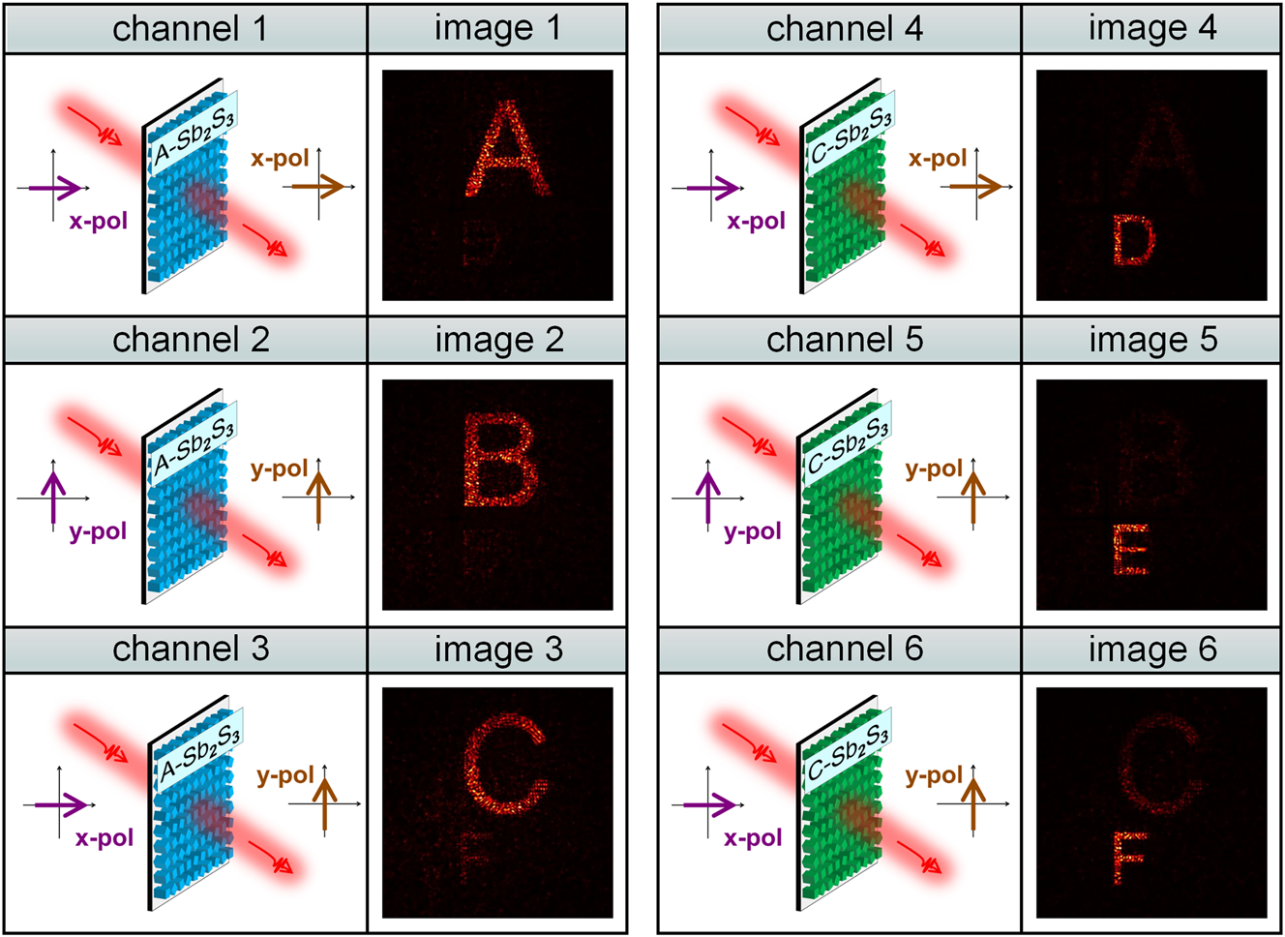
Reconstructed results of six distinct holographic images enabled by coordinated control over the crystallinity state of Sb_2_S_3_ and the incident/output polarization configuration. The designed metasurface supports polarization multiplexing across three orthogonal channels in both the A- and C-state, thereby achieving dynamic six-channel holographic switching within a single device.

As illustrated in Figure 5, precise and deterministic switching among all six holographic channels is realized through the coordinated control of the crystallinity state of Sb_2_S_3_ and the polarization configuration of the incident and transmitted light. The reconstructed holographic images exhibit high structural fidelity and agree well with the target images. Further improvements in image resolution and contrast can be achieved by increasing the number of addressable pixels and by adopting unit cells that yield more uniform transmission amplitudes. Compared with the previously discussed multifocal metalens, the holographic metasurface demonstrates a significantly reduced level of inter-state crosstalk. This reduction arises from the global nature of phase encoding produced by the GS algorithm, which introduces a holistic mismatch in the phase distribution when a hologram encoded for one material state is viewed under the other. As a result, undesired image reconstruction across states is intrinsically suppressed, and the optical information associated with each channel remains well isolated. For the holographic metasurface, the transmittances of the three channels are 41.9 %, 16.4 %, and 41.7 % in the A-state, and 13.2 %, 1.6 %, and 13.1 % in the C-state.

### 3.4 Progressive encoding via inter-state crosstalk

When the polarization channel is held constant, a degree of crosstalk between different crystallinity states inevitably arises due to the structural continuity. Complete suppression of this type of inter-state interference would necessitate the adoption of a more sophisticated iterative design framework, which would considerably increase the computational complexity and deviate from the principle of design simplification maintained throughout the present study. Further analysis reveals that, based on the existing design framework, further excluding (*L*, *W*) combinations with low $t_{x}^{\text{A}}$ values in Figure 4d will exert a direct and measurable influence on the extent of inter-state crosstalk (refer to Section 6 of the Supporting Information). In particular, the use of a higher transmittance threshold leads to a stronger retention of the optical response from A-state to C-state, thereby resulting in more pronounced inter-state evident crosstalk.

**Figure 6:**
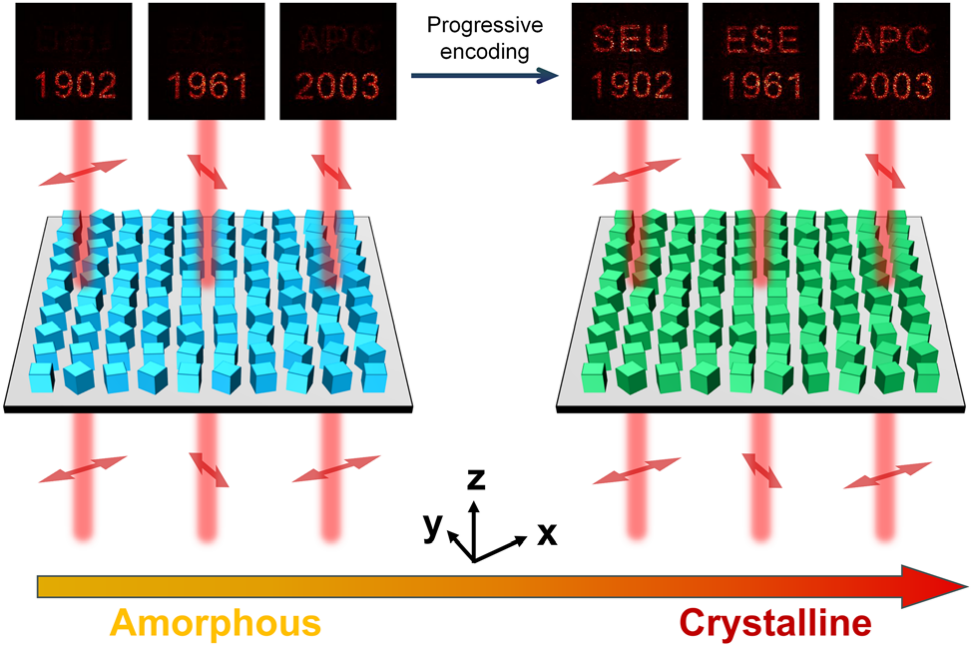
Demonstration of progressive holographic encoding enabled by inter-state optical imprinting. (a) In the A-state, the metasurface displays the numerical patterns “1902”, “1961”, and “2003”. (b) In the C-state, additional holographic images “SEU”, “ESE”, and “APC” appear superimposed, enabling layered information access through crystallinity switching.

Rather than viewing this residual overlap as a limitation, a complementary encoding strategy is adopted in which the optical imprint from the A-state is deliberately retained and utilized. By intentionally increasing the transmittance threshold in the A-state optimization process, the encoded optical information is carried forward into the C-state, where it is selectively superimposed upon newly introduced holographic features. This approach enables a form of progressive, layered information encoding, as illustrated in Figure 6. In the A-state, the metasurface reconstructs three distinct holographic images (namely the numerals “1902,” “1961,” and “2003”), each corresponding to one of the three orthogonal polarization channels. Upon switching to the C-state, these original images remain visible, while three additional holograms (labeled “SEU,” “ESE,” and “APC”) emerge as overlays on the existing set. In this manner, a dual-state encoding scheme is realized, through which six discrete pieces of information are sequentially embedded and retrieved from a single metasurface platform. This sequential encoding strategy holds significant potential for a variety of advanced photonic applications, such as optical data encryption, dynamic anti-counterfeiting, and reconfigurable information storage, especially in scenarios that require multi-level data representation and state-dependent optical switching based on material crystallinity.

## 4 Conclusions

In conclusion, this study proposes a unified design strategy for reconfigurable metasurfaces, enabling six independent, dynamically switchable phase modulation functionalities by simultaneously tailoring the polarization of light and the crystallinity-dependent optical response of phase-change material. Through the integration of a pixel-extension concept with a forward filtering algorithm, the proposed framework substantially expands the accessible degrees of freedom under dual-state constraints, thereby allowing for high-precision wavefront modulation within a single-layer architecture, which is validated by implementations of multifunctional metalens and metasurface holography. Moreover, rather than being treated as a limitation, the inter-state crosstalk from structural continuity is deliberately retained and strategically utilized for progressive information encoding, enriching functionality without added structural complexity. The proposed methodology exhibits strong potential for extensibility. Regarding the pixel-extension strategy, this study employed superpixels composed of diagonally adjacent nanofins, but alternative superpixel designs could also be explored. In terms of multi-state design, the current framework, initially applied to phase change metasurfaces, can naturally extend to multi-wavelength metasurfaces by treating material dispersion in a manner analogous to phase-change modulation. Furthermore, the incorporation of deep learning or other intelligent optimization techniques is expected to alleviate inter-channel crosstalk and enable polarization multiplexing in more than two states. This work highlights the capability of the proposed metasurface platform as a scalable, versatile foundation for next-generation adaptive photonic systems, secure optical processing, and hierarchical data storage applications.

## Supplementary Material

Supplementary Material Details
