# Supplementary material for: Dual-state six-channel polarization multiplexing in reconfigurable metasurfaces
Source: Nanophotonics. 2025 Nov 10;14(25):4583–93. doi: 10.1515/nanoph-2025-0403 (PMC12714064; doi:10.1515/nanoph-2025-0403)
Supplement: Supplementary file 1 — Supplementary Material Details [file j_nanoph-2025-0403_suppl_001.docx]

Supporting Information for

Dual-State Six-Channel Polarization Multiplexing in Reconfigurable Metasurfaces

Sujun Xie^1,†^, Tianxu Jia^1,2,†^, Xiaoyue Ma^3^, Bingjue Li^4^, Ruohu Zhang^1^, Binfeng Yun^1^, Hyeonsu Heo^2^, Nara Jeon^2^, Guanghao Rui^1,*^, and Junsuk Rho^2,5,6,7,*^

^1^Department of Optical Engineering, School of Electronic Science and Engineering, Southeast University, Nanjing 211189, Jiangsu, China

^2^Department of Mechanical Engineering, Pohang University of Science and Technology (POSTECH), Pohang 37673, Republic of Korea

^3^School of Electrical and Information Engineering, Tianjin University, Tianjin 300072, Tianjin China

^4^School of Mechanical Engineering, Southeast University, Nanjing 211189, Jiangsu, China

^5^Department of Chemical Engineering, Pohang University of Science and Technology (POSTECH), Pohang 37673, Republic of Korea;

^6^Department of Electrical Engineering, Pohang University of Science and Technology (POSTECH), Pohang 37673, Republic of Korea;

^7^POSCO-POSTECH-RIST Convergence Research Center for Flat Optics and Metaphotonics, Pohang, 37673 Republic of Korea

^†^These authors contributed equally to this work.

^*^Corresponding authors: E-mail: [ghrui@seu.edu.cn](mailto:ghrui@seu.edu.cn) (G. Rui), jsrho@postech.ac.kr (J. Rho).

## Section 1: Feasibility of three-channel phase modulation in the A-state

In this section, we reinterpret the complex variables in Eq. (4) of the main text as two-dimensional vectors in the complex plane and provide a geometric proof of the existence of valid solutions.

We begin by establishing the following geometric lemma. In the two-dimensional vector space, let *O* be the origin, $T_{x}^{A}$ and $T_{y}^{A}$ be two arbitrary vectors with magnitudes in the range [0, 1], and $\theta$ be any angle within [0, π]. Define the vectors $OA$, $OB$, $OC$, $OD$ as follows: $OA=T_{x}^{A}\cos^{2} \theta+ T_{y}^{A}\sin^{2} \theta$, $OB=T_{x}^{A}\sin^{2} \theta+ T_{y}^{A}\cos^{2} \theta$, $OC=T_{x}^{A}$，$OD=T_{y}^{A}$. We then show that the segment $AB$ is colinear with segment $CD$, he two segments share the same midpoint. This is proven as follows:

$$\begin{aligned} \frac{OA+OB}{2}=\frac{\left( T_{x}^{A}\cos^{2} \theta+T_{y}^{A}\sin^{2} \theta\right)+\left( T_{x}^{A}\sin^{2} \theta+ T_{y}^{A}\cos^{2} \theta\right)}{2}=\frac{OC+OD}{2},\#\text{(S1)} \end{aligned}$$

$$\begin{aligned} AB=T_{x}^{A}{(sin}^{2} \theta{- \cos}^{2} \theta)+T_{y}^{A}\left( \cos^{2} \theta-\sin^{2} \theta\right)=\left( OD-OC \right)\cos2\theta=CD\cdot C,\#\text{(S2)} \end{aligned}$$

where *C* is a constant. Equation (S1) demonstrates that $\vec{AB}$ and $\vec{CD}$ share the same midpoint, while Equation (S2) confirms that they are colinear.

Based on this lemma, the design process shown in Fig. S1 can be interpreted geometrically. The target phase values (𝜑₁, 𝜑₂, 𝜑₃) are first mapped to three directions in the 2D plane in Fig. S1(a), thereby forming a reference triangle, as shown in Fig. S1(b). By symmetrically extending the reference edge $L_{2}$ by a fixed length $L^{'}$ on both ends, an enlarged triangle ($\Delta EFG$) is constructed, as shown in Fig. S1(c, d). Notably, $L_{2}$ and $L_{4}$ are colinear and share the same midpoint. According to the above lemma, $T_{x}^{A}$ and $T_{y}^{A}$ can be assigned the directions of $EF$ and $EG$, respectively. By appropriately selecting $\theta$, one can always satisfy: $EP=T_{x}^{A}\cos^{2} \theta+ T_{y}^{A}\sin^{2} \theta$ and $EQ=T_{x}^{A}\sin^{2} \theta+ T_{y}^{A}\cos^{2} \theta$. Here, the triplet ($T_{x}^{A}$, $T_{y}^{A}$ and $\theta$) constitutes a valid solution to Eq. (4). Furthermore, since the extension length $L^{'}$ can be arbitrarily chosen, multiple geometric configurations exist that satisfy the same phase constraints, indicating that Eq. (4) admits infinitely many solutions. This geometric insight provides substantial design flexibility for constructing unit cells capable of simultaneously modulating all three polarization channels in the A-state.


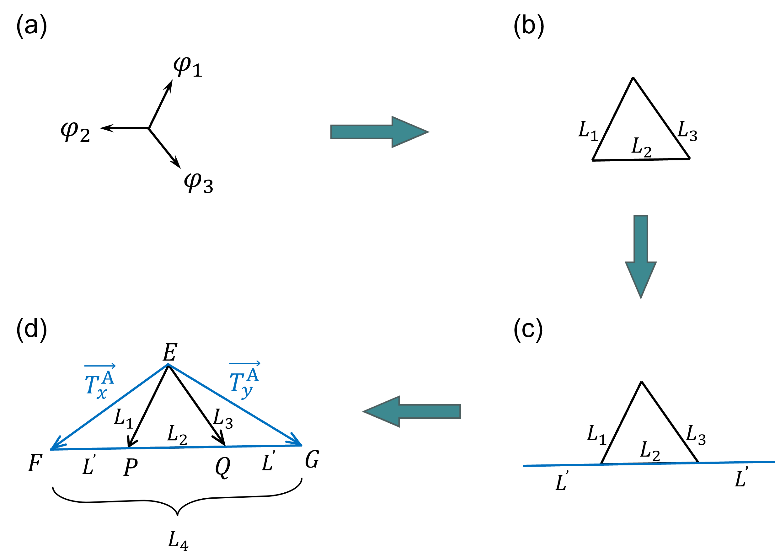


**Figure S1**: Geometric interpretation of the three-channel phase modulation design.

## Section 2: Limitation of Phase Modulation in Uniform-Pixel PCM Metasurfaces

In this section, we analyze reconfigurable metasurfaces in which the pixel configuration remains unchanged before and after the material phase transition. Each nanofin is treated as a single pixel, and its optical response is described by the Jones matrix defined in Eq. 3 of the main text.

Upon undergoing a phase transition, the birefringent transmission coefficients of the nanofin change to $T_{x}^{C}$ and $T_{y}^{C}$, while the orientation angle $\theta$ remains fixed. The corresponding Jones matrix in the crystalline state becomes:

$$\begin{aligned} J_{C}=\left[ \begin{matrix} T_{x}^{C}\cos^{2} \theta+T_{y}^{C}\sin^{2} \theta& \left( T_{x}^{C}-T_{y}^{C} \right)\sin\theta\cos\theta\\ \left( T_{x}^{C}-T_{y}^{C} \right)\sin\theta\cos\theta& T_{x}^{C}\sin^{2} \theta+T_{y}^{C}\cos^{2} \theta\end{matrix} \right].\#\text{(}\text{S}\text{3)} \end{aligned}$$

Since $\theta$ is fixed after fabrication, only two independent parameters ($T_{x}^{C}$ and $T_{y}^{C}$) can be adjusted. To investigate the feasibility of multi-channel modulation under these constraints, we consider two arbitrary target phase values $\varphi_{4}$ and $\varphi_{5}$, and assign them to two distinct elements of the Jones matrix, denoted as $J_{C1}$ and $J_{C2}$, where $J_{C1}, J_{C2}\in\left\{ J_{C}\left( 1,1 \right),J_{C}\left( 1,2 \right),J_{C}\left( 2,2 \right) \right\}$ and $J_{C1}\neq J_{C2}$. Solving the system: $\varphi_{4}={arg(J}_{C1})$and $\varphi_{5}={\arg(J}_{C2})$ for multiple representative parameter sets, we find that despite having the same number of variables as constraints, the system generally does not admit a solution. This is due to the intrinsic coupling of matrix elements under the given birefringent structure and fixed $\theta$. Nevertheless, it is worth noting that the off-diagonal elements of $J_{C}$ are independent of $\theta$, and depend only on the relative values of $T_{x}^{C}$ and $T_{y}^{C}$. This observation suggests that additional degrees of phase modulation may be introduced specifically in the cross-polarized channels after the phase transition. As a result, in the absence of the pixel-extension strategy proposed in the main text, a metasurface composed of uniform individual nanofins is fundamentally limited to supporting at most four independently controlled polarization channels. This underscores the necessity of the superpixel-based design approach developed in this work, which enables full six-channel modulation.

## Section 3: Feasibility of three-channel phase modulation in the C-state

This section examines the solvability of Eq. (6) in the main text, which represents a nonlinear system over the complex field. Unlike the geometric vector approach used for the A-state analysis in Section S1, here we employ the implicit function theorem and Jacobian rank conditions to assess the feasibility of independent phase control. Essentially, this corresponds to determining the local solvability of an underdetermined nonlinear system with four degrees of freedom and three constraints.

Let $\tilde{\boldsymbol{T}}=\left[ T_{x1}^{C}, T_{y1}^{C},T_{x2}^{C},T_{y2}^{C} \right]^{T}\in\mathbb{R}^{4},$ representing four parameters associated with the birefringent responses of two nanofins in the C-state. The three independent complex-valued elements of the Jones matrix $J_{C}$ can be expressed as: $f_{1}\left( \tilde{\boldsymbol{T}} \right)=J_{C11}\left( \tilde{\boldsymbol{T}} \right)$, $f_{2}\left( \tilde{\boldsymbol{T}} \right)=J_{C12}\left( \tilde{\boldsymbol{T}} \right)$, $f_{3}\left( \tilde{\boldsymbol{T}} \right)=J_{C22}\left( \tilde{\boldsymbol{T}} \right)$. Our objective is to determine whether the phase components $arg(f_{1})$, $arg(f_{2})$, $arg(f_{3})$ can be independently controlled through appropriate selection of $\tilde{\boldsymbol{T}}$. For any arbitrary phase triplet $\left( \alpha, \beta, \gamma\right)\in\left[ 0, 2\pi\right]^{3}$, we define a set of real-valued functions:

$$\begin{aligned} \begin{aligned} F_{1}\left( \tilde{\boldsymbol{T}} \right)=\mathrm{Im}\left[ f_{1}\left( \tilde{\boldsymbol{T}} \right) \right]-\tan\left( \alpha\right)\cdot\mathrm{Re}\left[ f_{1}\left( \tilde{\boldsymbol{T}} \right) \right]\# \\ F_{2}\left( \tilde{\boldsymbol{T}} \right)=\mathrm{Im}\left[ f_{2}\left( \tilde{\boldsymbol{T}} \right) \right]-\tan\left( \beta\right)\cdot\mathrm{Re}\left[ f_{2}\left( \tilde{\boldsymbol{T}} \right) \right] \\ F_{3}\left( \tilde{\boldsymbol{T}} \right)=\mathrm{Im}\left[ f_{3}\left( \tilde{\boldsymbol{T}} \right) \right]-\tan\left( \gamma\right)\cdot\mathrm{Re}\left[ f_{3}\left( \tilde{\boldsymbol{T}} \right) \right] \end{aligned}.\#\text{(S4)} \end{aligned}$$

These functions define a mapping *F*: $\mathbb{R}^{4}$ → $\mathbb{R}^{3}$ given by:

$$\begin{aligned} F\left( \tilde{\boldsymbol{T}} \right)=\left[ \begin{matrix} F_{1}\left( \tilde{\boldsymbol{T}} \right) \\ F_{2}\left( \tilde{\boldsymbol{T}} \right) \\ F_{3}\left( \tilde{\boldsymbol{T}} \right) \end{matrix} \right].\#\text{(}\text{S5}\text{)} \end{aligned}$$

Solving $F\left( \tilde{\boldsymbol{T}} \right)=0$ is equivalent to assigning prescribed phase values to the three selected elements of $J_{C}$. Suppose there exists a solution $\tilde{\boldsymbol{T}_{\boldsymbol{0}}}\in\mathbb{R}^{4}$ such that $F\left( \tilde{\boldsymbol{T}_{\boldsymbol{0}}} \right)=0$, Let $J_{F}\left( \tilde{\boldsymbol{T}_{\boldsymbol{0}}} \right)\in\mathbb{R}^{3}ˣ^{4}$denote the Jacobian matrix of *F* evaluated at $\tilde{\boldsymbol{T}_{\boldsymbol{0}}}$:

$$\begin{aligned} J_{F}\left( \tilde{\boldsymbol{T}_{\boldsymbol{0}}} \right)=\left[ \frac{\partial F_{i}}{\partial\tilde{T_{j}}}\left( \tilde{\boldsymbol{T}_{\boldsymbol{0}}} \right) \right]_{i=1,2,3}^{j=1,2,3,4}.\#\text{(}\text{S6}\text{)} \end{aligned}$$

If $\mathrm{rank}\left[ J_{F}\left( \tilde{\boldsymbol{T}_{\boldsymbol{0}}} \right) \right]=3$, then by the implicit function theorem, the solution $\tilde{\boldsymbol{T}_{\boldsymbol{0}}}$ is locally stable, and small perturbations in the phase values ($\delta\alpha, \delta\beta, \delta\gamma$) correspond to continuous solutions $\tilde{\boldsymbol{T}}$ within a neighborhood of $\tilde{\boldsymbol{T}_{\boldsymbol{0}}}$. This implies local surjectivity of the mapping from $\tilde{\boldsymbol{T}}$ to ($arg(f_{1})$, $arg(f_{2})$, $arg(f_{3})$), and thus local controllability of the three phase components with respect to the four tunable parameters. It remains to verify that the Jacobian matrix $J_{F}$ generically has full row rank. Each function $f_{i} (i=1,2,3)$ is a linear combination of terms proportional to $t_{k}e^{i\varphi_{k}}$, with distinct real-valued coefficients that depend on the rotation angles $\theta$₁ and $\theta$₂. These coefficients are non-degenerate for generic values of $\theta$₁ and $\theta$₂, particularly when $\sin\theta_{i}\cos\theta_{i}\neq0 (i=1,2)$. As a result, the real and imaginary parts of $f_{i}$ vary independently with respect to the four components of $\tilde{\boldsymbol{T}}$, implying that $J_{F}$ achieves full rank (=3) almost everywhere in parameter space. In degenerate cases such as $\theta$₁ = 0 or $\theta$₂ = 0, rank deficiency may arise. However, these cases correspond to a measure-zero subset and do not affect the generic behavior. Therefore, for generic choices of $\theta$₁ and $\theta$₂, the four phase parameters embedded in $\tilde{\boldsymbol{T}}$ provide sufficient freedom to independently control the phases of all three complex elements of the symmetric Jones matrix $J_{C}$.

## Section 4: Design details of the proposed forward filtering algorithm

To illustrate how the global layout is generated, a metasurface composed of 4 × 4 nanofins is adopted as an example. As shown in Fig. S2, this structure corresponds to 4 × 4 effective pixels in the A-state and 2 × 2 effective pixels in the C-state. First, following the superpixel grouping method described in the main text, the required A-state phases are divided into two sets, which corresponds to the “split” step in Fig. S2. Next, for each superpixel formed by two diagonally adjacent nanofins, the proposed forward design strategy is used to determine the geometric parameters of these two nanofins. Although the two superpixel groups share the same C-state phase profiles (𝜑_4_, 𝜑_5_, 𝜑_6_), their design procedures are independent, as indicated by the blue and green arrows in Fig. S2. Finally, in the “combine” step of Fig. S2, the optimal solutions of all pixels are assembled into the complete metasurface layout.


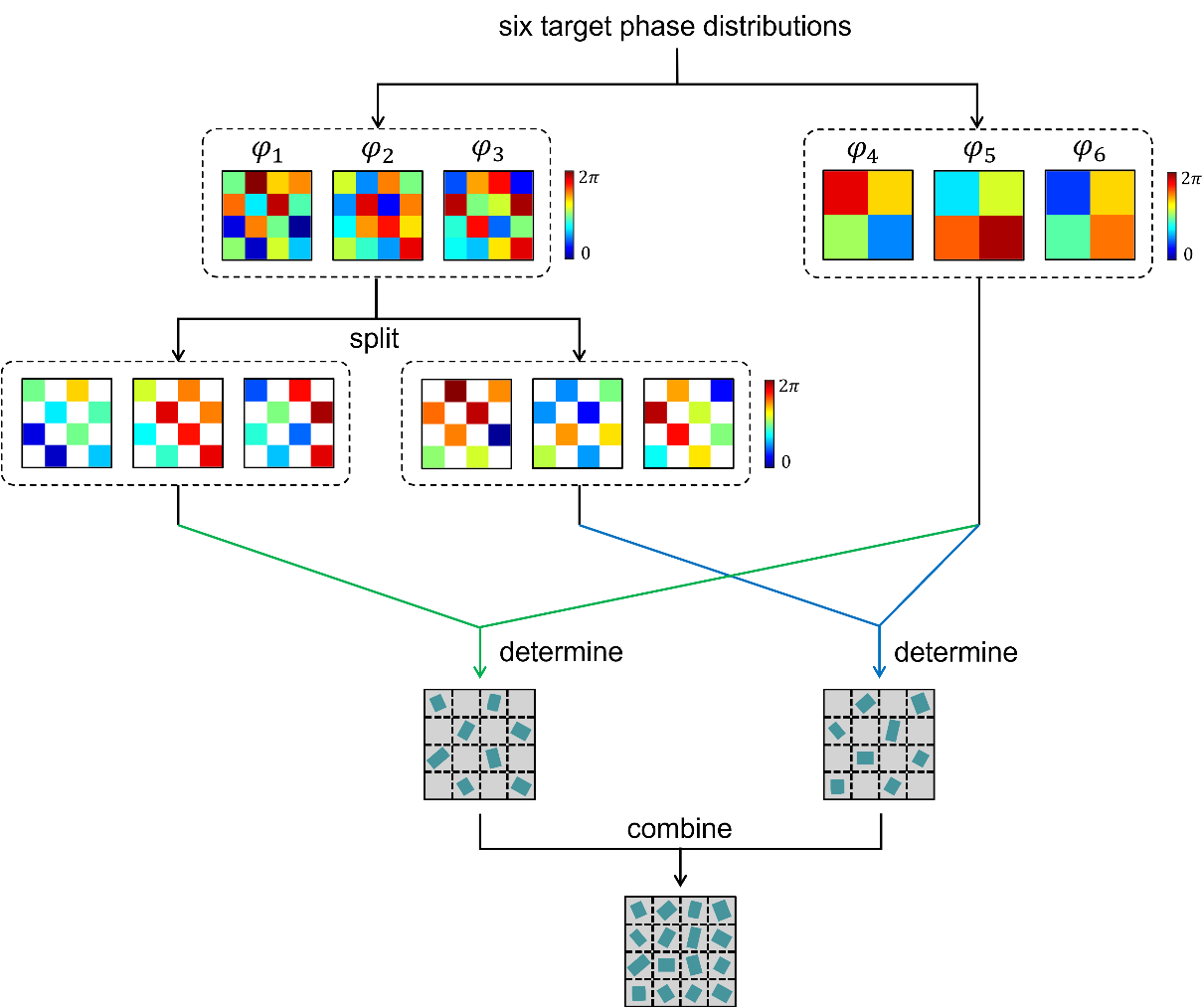


**Figure S2**: Global design process for metasurfaces. 𝜑_1_, 𝜑_2_ and 𝜑_3_ denote the three phase components of the A-state target Jones matrix JA-target. 𝜑_4_, 𝜑_5_ and 𝜑_6_ denote the three phase components of the C-state target Jones matrix JC-target. Only phase modulation is considered, so the amplitude term of the Jones matrix is omitted.

## Section 5: Design details of the mutifocal metalens

To verify the effectiveness of the proposed dual-state design strategy, we compare the theoretical phase profiles with the corresponding realized phase distributions obtained through full-wave simulations, as illustrated in Fig. S3.


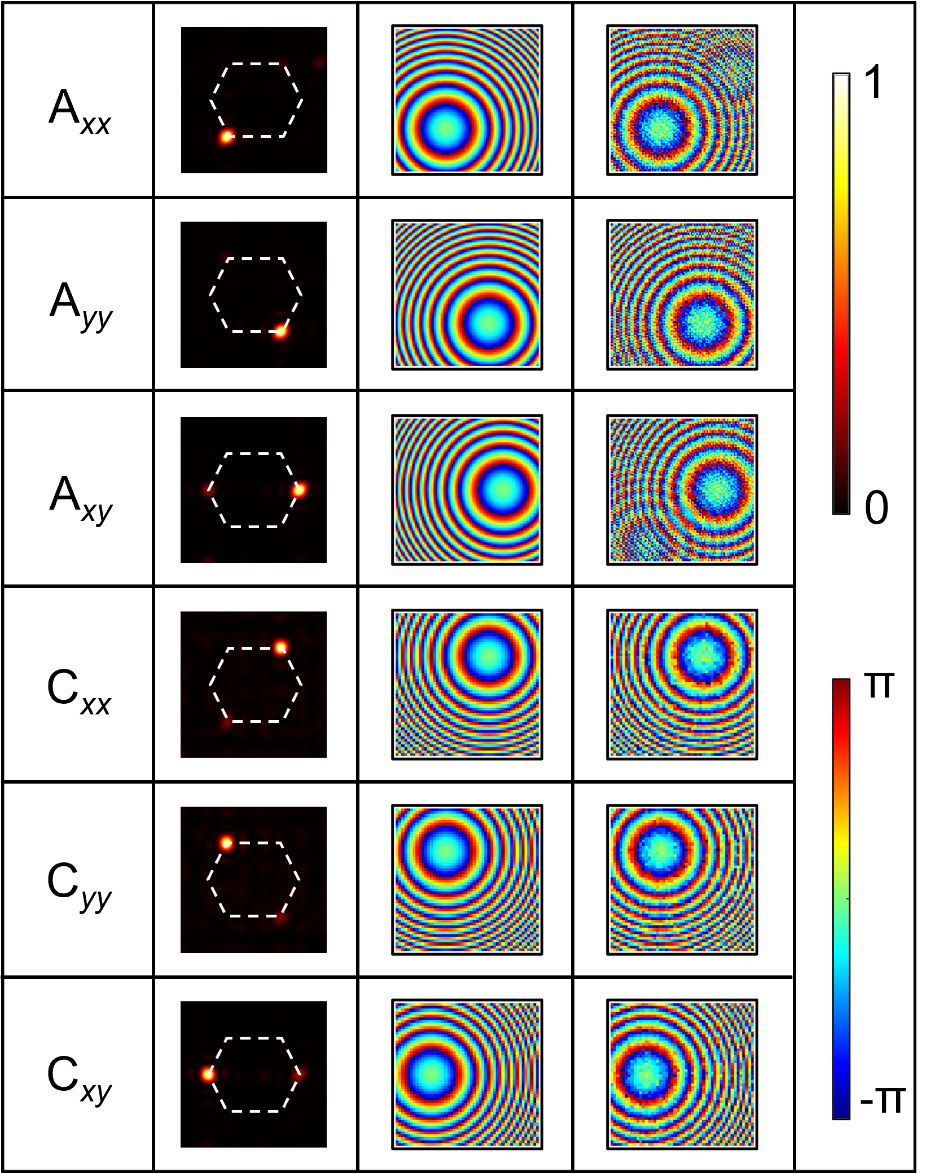


**Figure S3**: Simulated focal intensity distributions (left), target phase profiles (middle), and realized phase maps (right) for the six polarization–crystallinity channels (A*_xx_*, A*_yy_*, A*_xy_*, C*_xx_*, C*_yy_*, C*_xy_*) of the reconfigurable multifocal metalens.

Each of the six optical channels operates independently and is labeled as A*_xx_*, A*_yy_*, A*_xy_*, C*_xx_*, C*_yy_*, and C*_xy_*, where the leading character (A or C) denotes the material state and the subscripts represent the polarization states of the incident and transmitted light, respectively. For instance, A*_xx_* refers to the configuration in which *x*-polarized incident light yields *x*-polarized transmitted light when the metasurface is in the amorphous state. Across all six channels, strong agreement is observed between the target phase profiles and the realized distributions, demonstrating the fidelity of the proposed design. Minor discrepancies arise primarily from the discretization of structural parameters and the quantization of phase values during fabrication-oriented mapping; however, these deviations do not substantially affect the optical performance, particularly in terms of focal spot quality. These results confirm that accurate and independent phase modulation is achieved in all channels, thereby validating the practicality and versatility of the proposed multifunctional metasurface platform.

## Section 6: Controlling crosstalk intensity via transmittance thresholding

In the design of high-efficiency metasurfaces, it is common practice to implement a transmission-based filtering procedure, whereby nanofin geometries with transmittance values below a specified threshold are excluded from the design database. However, due to the intrinsically higher optical loss in crystalline Sb₂S₃ at the design wavelength of 633 nm, applying such a threshold uniformly to both material states would significantly reduce the number of viable (*L–W*) combinations in the crystalline state, thereby limiting the achievable phase coverage and design flexibility. Fortunately, as shown in the upper-left panel of Fig. S4(c), the amorphous state of Sb₂S₃, characterized by a low extinction coefficient, supports a wide range of (*L–W*) combinations with relatively high transmittance. This permits the introduction of a lower-bound transmittance threshold $t_{min}^{A}$ during A-state optimization to enhance performance without sacrificing too much design space.

The A-state transmittance threshold governs the trade-off between functional independence and intentional crosstalk. A higher threshold promotes retention of A-state features in the C-state and enables progressive information encoding. A lower threshold suppresses inter-state crosstalk and preserves distinct, independent functions in the two material states. Because crosstalk is explicitly regulated under this scheme, even deliberate increases do not lead to functional ambiguity between states.


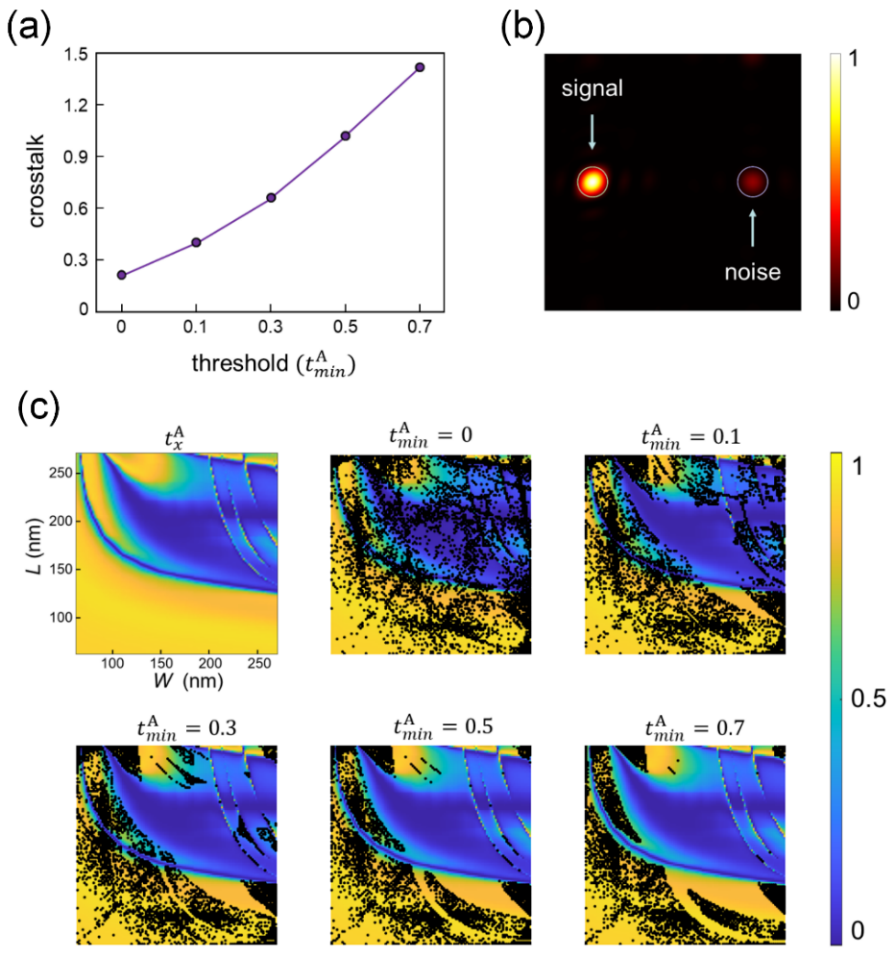


**Figure S4**: Comparison of design strategies and optical responses under varying A-state transmittance thresholds. (a) Quantified average inter-state crosstalk levels across the three polarization channels. (b) Definition of signal and noise: optical intensity at intended target positions is treated as signal, whereas responses appearing at corresponding positions under the opposite material state are defined as noise. (c) Distribution of (*L–W*) combinations retained after applying different $t_{min}^{A}$ thresholds during the A-state design.

We then investigate how varying $t_{min}^{A}$ influences the system’s optical behavior. As illustrated in Fig. S4(a), increasing $t_{min}^{A}$ from 0 to 0.7 leads to a monotonic rise in the functional leakage from the A-state into the C-state, which is defined as inter-state crosstalk and quantitatively assessed via the average noise-to-signal ratio across all polarization channels, as shown in Fig. S4(b). Notably, once $t_{min}^{A}$ exceeds 0.5, the leaked residual functionality from the A-state begins to dominate over the intentional functionality in the C-state, with this effect becoming increasingly pronounced at $t_{min}^{A}$ = 0.7. This behavior forms the theoretical foundation of our progressive information encoding strategy, in which residual optical features from the A-state are intentionally retained to realize layered information representation across material states. Furthermore, as shown in Fig. S4(c), we examine the spatial distribution of selected (*L–W*) regions under various threshold settings. It is evident that higher thresholds significantly constrain the designable area within the parameter space, which directly leads to distinct differences in the resulting optical functionalities.
